# Tom70-regulated mitochondrial biogenesis via TFAM improves hypoxia-induced dysfunction of pulmonary vascular endothelial cells and alleviates hypoxic pulmonary hypertension

**DOI:** 10.1186/s12931-023-02631-y

**Published:** 2023-12-13

**Authors:** Lei Ma, Yanxia Wang, Xiaoqian Li, Zefang Wang, Bo Zhang, Ying Luo, Yousheng Wu, Zhichao Li, Wen Niu

**Affiliations:** 1 Department of Anesthesiology, Second Affiliated Hospital of Xi’an Jiaotong University, 157 Xiwu Street, Xi’an, 710004 People’s Republic of China; 2 Department of Pathology, Xijing Hospital and School of Basic Medicine, Air Force Medical University, 169 Changle Western Street, Xi’an, 710032 People’s Republic of China; 3 Department of Cardiology, Second Medical Center and National Clinical Research Center for Geriatric Diseases, Chinese PLA General Hospital, 28 Fuxing Street, Beijing, 100853 People’s Republic of China; 4 Department of Basic Medicine, Graduate School, Chinese PLA General Hospital, 28 Fuxing Street, Beijing, 100853 People’s Republic of China; 5 Department of Physiology and Pathophysiology, School of Basic Medicine, Air Force Medical University, 169 Changle Western Street, Xi’an, 710032 People’s Republic of China; 6 National Demonstration Center for Experimental Preclinical Medicine Education, Air Force Medical University, 169 Changle Western Street, Xi’an, 710032 People’s Republic of China; 7 Key Laboratory of Resource Biology and Biotechnology in Western China, Ministry of Education, School of Medicine, Northwest University, 229 Taibai North Street, Xi’an, 710069 People’s Republic of China

**Keywords:** Hypoxic pulmonary hypertension, Pulmonary vascular endothelial cells, Translocase of outer mitochondrial membrane, Mitochondrial biogenesis

## Abstract

**Background:**

Hypoxic pulmonary hypertension (HPH) is a common type of pulmonary hypertension and characterized by pulmonary vascular remodeling and constriction. A large number of studies have shown that pulmonary vascular endothelial cells (PVECs) dysfunction plays an important role in the initiation and development stages of HPH, but the mechanism of PVECs dysfunction after hypoxia remains unclear. In this study, we explored the exact mechanism of PVECs dysfunction after hypoxia.

**Methods:**

In vitro, we used primary cultured PVECs hypoxia model to mimic HPH injury. We detected the expressions of mitochondrial biogenesis markers, mitochondrial transcription factor A (TFAM) level inside mitochondria, mitochondrial quantity and function, and the components expressions of translocase of outer mitochondrial membrane (TOM) at 24 h after hypoxia. To explore the effects of Tom70 on mitochondrial biogenesis and functions of PVECs after hypoxia, Tom70 overexpression adenovirus was constructed, and the expressions of mitochondrial biogenesis markers, TFAM level inside mitochondria, mitochondrial quantity and function, and the functions of PVECs were detected. And in vivo, we used cre-dependent overexpression adenovirus of Tom70 in the Cdh5-CreERT2 mouse model of HPH to verify the role of upregulating PVECs Tom70 in improving HPH.

**Results:**

Hypoxia obviously increased the expressions of mitochondrial biogenesis markers for PGC-1α, NRF-1 and TFAM, but reduced the content of TFAM in mitochondria and the quantity and functions of mitochondria. In addition, only Tom70 expression among the TOM components was significantly decreased after hypoxia, and up-regulation of Tom70 significantly increased the content of TFAM in mitochondria of PVECs by transporting TFAM into mitochondria after hypoxia, enhanced the quantity and functions of mitochondria, improved the functions of PVECs, and ultimately alleviated HPH.

**Conclusion:**

The findings of present study demonstrated that hypoxia induced the decreased expression of Tom70 in PVECs, reduced the mitochondrial biogenesis-associated TFAM protein transporting into mitochondria, inhibited mitochondrial biogenesis, caused PVECs injury, and prompted the formation of HPH. However, up-regulation of Tom70 abolished the hypoxia-induced injurious effects on PVECs and alleviated HPH.

**Supplementary Information:**

The online version contains supplementary material available at 10.1186/s12931-023-02631-y.

## Background

Hypoxic pulmonary hypertension (HPH) is the leading cause of disability and death in chronic mountain sickness (CMS) and chronic obstructive pulmonary disease (COPD), which brings great economic and social burden to the patients and countries [1, 2]. At present, the vasodilators are the main drug for HPH, however, the therapy effect is not satisfactory with a 1-year mortality rate of up to 15% [3, 4]. Therefore, it is urgent to elucidate the mechanism of HPH formation and explore the effective therapeutic targets for HPH.

HPH is a disease characterized by early pulmonary vasoconstriction and late pulmonary vascular wall remodeling, with a complex pathogenesis and multiple factors involved in the pathological process [5]. In recent years, a large number of studies have shown that pulmonary vascular endothelial dysfunction plays an important role in the initiation and development stages of HPH [6–8]. Pulmonary vascular endothelium not only has physiological barrier function, but also acts as an endocrine organ, secreting various vasoactive substances to regulate vascular tension [9]. After hypoxia, the endothelial barrier function is impaired, causing a large number of growth factors in the blood to directly act on the smooth muscle layer, and leading to a large proliferation of smooth muscle cells [10]. Moreover, hypoxia disrupts the secretion function of endothelial cells, with the reduced production of vasodilators such as nitric oxide (NO), and the increased secretion of vasoconstrictors such as endothelin (ET-1), which lead to abnormal constriction of pulmonary blood vessels and subsequent structural reconstruction of pulmonary blood vessels [11–13]. In sum, the dysfunction of pulmonary vascular endothelial cells (PVECs) exists in the entire pathophysiological process of HPH, and is a crucial mechanism for the formation of HPH. However, the exact mechanism of PVECs dysfunction after hypoxia is still obscure.

Mitochondria, as the energy factory of cells, participate in physiological processes such as oxidative stress, apoptosis, and calcium homeostasis regulation. In addition, mitochondrial dysfunction is believed to be a key pathological characteristic of cellular injury [14]. Several studies have shown that mitochondrial injury is the key mechanism for dysfunction of PVECs after hypoxia [15, 16]. After hypoxia, the antioxidant capacity of mitochondria in PVECs decreases, and the production of reactive oxygen species (ROS) and free radicals increases, which lead to the reduction in mitochondrial quantity and function, ultimately leading to PVECs injury [17]. Therefore, regulating mitochondrial function is considered an effective therapeutic strategy to improve the function of PVECs and reduce their apoptosis after hypoxia.

In this study, we used hypoxia model in primary cultured PVECs to mimic PVECs injury induced by HPH, and investigated the mechanism of mitochondrial injury in PVECs after hypoxia. Moreover, we also used HPH model in mice to explored the targeted intervene methods for improving PVECs and mitochondrial function after hypoxia, and eventually alleviating HPH.

## Methods

### Animals and cells

All animal procedures in this study were approved by the Ethics Committee for Animal Experimentation of Fourth Military Medical University and were conducted according to the Fourth Military Medical University Guidelines for Animal Experimentation (Xi’an, China). Male Sprague–Dawley (SD) rats were provided by the Experimental Animal Center of Fourth Military Medical University. Rat primary PVECs were obtained by tissue-piece culturing method. Under sterile conditions, take lung tissue from adult SD rats, cut lung tissue 2 mm from the edge of the lung lobe, and cut it into a tissue block with a size of about 1 mm^3^. Lay it flat on the bottom of the culture bottle. After placing the culture bottle vertically, add endothelial cell specific culture medium (M200 medium + 2 ml LSGS growth factor/100 ml culture medium + 10% fetal bovine serum). Place the tissue block face up and incubate in an incubator for about 3 h. After the tissue block is firmly attached, gently turn the culture bottle upside down and continue cultivating for 24–48 h. To obtain purified endothelial cells, blow off the tissue mass at this time to prevent fibroblasts from crawling out, and continue to culture for 5–7 days. After trypsin digestion, flow cytometry sorting and passage were performed, and the third generation cells were taken. The classification and purity of PVECs was further identified respectively by the immunohistochemical staining of PVECs marker CD31 and flow cytometry.

The Cdh5-CreERT2 mice were purchased from Cyagen (Suzhou, China). To obtain HPH mice, male mice were housed in a hypobaric hypoxia chamber depressurized to 380 mmHg (10% O_2_) for 4 weeks. Age-matched mice were housed in room air (21% O_2_) accordingly as controls.

### Experimental protocol

In the first part of in vitro experiment, the PVECs were grouped as follows: (1) Normoxia, (2) Hypoxia. The PVECs were maintained under normoxia (21% O_2_) or hypoxia conditions (5% O_2_) for 24 h, and subsequently processed for the next detection. In the other parts of in vitro experiment, the PVECs were grouped as follows: Normoxia, Hypoxia and Adv-Tom70 + Hypoxia groups. The PVECs in Adv-Tom70 + Hypoxia group were cultured in the medium containing Tom70 overexpression adenovirus for 24 h, and subsequently maintained under hypoxia conditions (5% O_2_) for 24 h. All the PVECs of three groups were extracted respectively 24 h after normoxia (21% O_2_) or hypoxia (5% O_2_) treatment and processed for subsequent experiments.

The cre-dependent overexpression adenovirus of Tom70 was purchased from BrainVTA (Wuhan, China) and was given by intratracheal instillation in Cdh5-CreERT2 mice, selectively upregulating the expression of Tom70 in PVECs. The overexpression efficiency was verified by the methods of immunoelectron microscopy and immunofluorescence 4 weeks after intratracheal instillation. In vivo experiment, the mice were grouped as follows: Normoxia, Hypoxia and Adv-Tom70 + Hypoxia groups. The mice in Adv-Tom70 + Hypoxia group received Tom70 overexpression adenovirus by intratracheal instillation, and subsequently housed in a hypobaric hypoxia chamber for 4 weeks. All the mice of three groups were processed respectively 4 weeks after normoxia (21% O_2_) or hypoxia (10% O_2_) treatment for subsequent experiments.

### Flow cytometry

To identify the purity of cells, PVECs were detached using 0.02% EDTA, and stained with FITC-conjugated CD31 primary antibody (10 μL per 90 μL of resuspended cells, Abcam Cambridge, UK). Stained cells were analyzed with FACS flow-cytometer (BD, USA).

### mtDNA level

Real-time PCR measures the amount of mitochondrial DNA (mtDNA) replication. The Cyt b gene is a conserved sequence encoded by mtDNA, and the content of Cyt b is measured to reflect mtDNA. The primer design was as follows: Cyt b primer sequences: 5ʹ-TCCACTTCATCCCATTC-3ʹ, the reverse primer: 5ʹ-CTGCGTCGGAGTTTAATCCT-3ʹ; the internal reference gene GAPDH has a primer sequences of 5ʹ-ACAGCAACAGGGTGGGTGGAC-3ʹ, and the reverse primer of 5ʹ-TTTGAGGGTGCAGCGAACTT-3ʹ. The amplification results are analyzed using the analysis program provided by the fluorescence PCR instrument, and then their corresponding Ct values are calculated ΔCt = CtTARGE − CtGAPDH. The value of target gene = 2 − ΔCt value.

### Western blot analysis

After the treatments, all the cells were collected. And the cell protein concentration was evaluated by using the Bradford method, and Western blot analysis was performed as we previously described [18]. The following primary antibodies were used: anti-PGC-1α (1:3000; Cell Signaling, USA), anti-NRF-1 (1:1000; Abcam Cambridge, UK), anti-TFAM (1:1000; Cell Signaling, USA), anti-COX IV (1:500; Cell Signaling, USA), anti-Tom70 (1:1000, Abcam Cambridge, UK), and anti-GAPDH (1:1000, CWBIO, China). Appropriate horseradish peroxidase-conjugated secondary antibodies (1:10,000, CWBIO, China) were used. Image analysis was accomplished with the computerized analysis software (Bio-Rad Laboratories, USA). All original, full-length gel and blot images were presented in Additional file 1.

### Immunohistochemistry

The cells were seeded in confocal microscope specific cell culture plate at a density of 1 × 10^5^ cell/well. After the treatments, the cells were fixed with 4% paraformaldehyde for 30 min. After the washes, the cells or lung tissue sections were exposed respectively to primary antibodies (CD31, 1:1000; Tom70, 1:100; TFAM, 1:100) overnight at 4 °C. The cells or lung tissue sections were washed three times with PBS, followed by a 30-min exposure of FITC/Cy3-labled secondary antibody (1:200) in darkness or horseradish peroxidase-conjugated secondary antibody (1:10,000). Then, the cells or lung tissue sections were observed by using a confocal microscope (Olympus, Japan).

### Transmission electron microscopy

After all the treatments, the cell culture medium was removed, and washed with PBS for three times. Then the PVECs were fixed with PBS solution containing 4% paraformaldehyde for 30 min. After the fixation, the PVECs were observed with a JEM-2000EX transmission electron microscope (JEOL, Japan).

### Tom70 overexpression in vitro

Design primers based on the cDNA sequence of Tom70, amplify Tom70 cDNA from the total mRNA of cells using RT-PCR technology. Then sequence the PCR products, and clone the correctly sequenced Tom70 cDNA into an adenovirus expression vector, which is then transferred into pulmonary vascular endothelial cells.

### Mitochondrial functions detection

Active mitochondria are obtained by using a mitochondrial extraction kit (Qiagen, Germany), and the mitochondrial activity and purity are detected with Janus green B staining and transmission electron microscopy.

The intracellular ATP levels were detected by using an ATP assay kit (Beyotime, China) according to the instructions of the manufacture and the luminance was detected by using a fluorescence microplate reader (Bio-Rad, USA). Data were normalized to the normoxia group and expressed as percentage of the normoxia group.

Oxygen consumption rate (OCR) and extracellular acidification rate (ECAR) were measured respectively by using Seahorse XF cell mitochondrial stress test kit and Seahorse XF glycolysis stress test kit (Agilent Technologies, USA) according to the manufacturer’s instructions and were analyzed by the Seahorse extracellular flux (XF96) analyzer (Agilent Technologies, USA). PVECs (2 × 10^4^ cells/well) were seeded on Seahorse cell culture microplates and received corresponding treatments. For OCR test, oligomycin (2 uM), FCCP (2 uM), and antimycin A (2 uM) were delivered into the appropriate ports sequentially. For ECAR test, glucose (10 mM), oligomycin (2 uM) and 2-deoxy-d-glucose (2-DG, 50 mM) were delivered into the appropriate ports sequentially. After calibration, OCR and ECAR were measured every 8 min for 96 min, and were automatically calculated using the Seahorse XF96 software.

Mitochondrial swelling was measured as an indication of mitochondrial permeability transition pore (MPTP) opening according to our previous description [18]. Briefly, 1 mg of isolated mitochondria was suspended in 1 ml of respiration buffer plus 10 mM succinate. After a 5-min preincubation at 36 °C and baseline measurement, CaCl_2_ (40 nM) was added. The absorbance at 520 nm (A_520_) was measured with a spectrophotometer (Tecan, Switzerland) at 36 °C and a change of absorbance at 10 min following the addition of CaCl_2_ indicated mitochondrial swelling.

Mitochondrial membrane potential (MMP) was evaluated by using the JC-1 fluorescent probe (Sigma-Aldrich, USA) according to the instructions of the manufacture. The change of MMP can be detected through fluorescence color conversion by using a confocal microscope (Olympus, Japan).

Mitochondrial complex I and IV were evaluated at 30 °C spectrophotometrically as the instructions of the manufacture. The mitochondria in each group were isolated and purified, and the detection wavelengths of complex I (NADH dehydrogenase), complex IV (cytochrome C oxidase) were 340 nm and 550 nm, respectively.

### Cell viability evaluation

PVECs were cultured in 96-well plates at a density of 1 × 10^5^ cells/well. After the treatments, cell viability was evaluated by the WST-8 dye. WST-8 dye (20 μl) was added into each well and after incubation at 37 °C for 3 h. The absorbance was measured at a wavelength of 450 nm using a spectrophotometer (Tecan, Switzerland).

### Lactate dehydrogenase (LDH) release measurement

PVECs were cultured in 24-well plates at a density of 2 × 10^5^ cells/well. After the treatments, supernatants were removed from each well to evaluate lactate dehydrogenase (LDH) levels using the LDH kit according to the manufacturer’s instructions. The absorbance of the sample blank, standard, and standard blank was measured at the same time. LDH activity was calculated according to the following formula:

$${\text{LDH activity}}\left( {{\text{U}}/{\text{L}}} \right) = \left[ {\frac{{{\text{sample OD}} - {\text{sample blank OD}}}}{{{\text{standard OD}} - {\text{standard blank OD}}}}} \right] \times {2} \times {1}000{\text{ U}}/{\text{L}}.$$

### Cell apoptotic rate assay

The rate of apoptosis in PVECs was evaluated by flow cytometry (BD, USA). Briefly, cells were cultured in a 6-well plate at a density of 5 × 10^5^ cells/well. After treatment, the cells were harvested by centrifugation at 1000 rpm for 5 min. After two washes with PBS, the cells were resuspended in binding buffer at a density of 1 × 10^6^ cells/ml. Then, 5 µl of FITC-conjugated anti-annexin-V staining antibody and 2 µl of propidium iodide solution were added to 100 µl of the binding buffer. After thorough mixing, the cells were incubated for 15 min at room temperature in dark, and the apoptotic rate was assessed.

### Intracellular reactive oxygen species (ROS) detection

Intracellular ROS was detected by ROS detection reagents utilizing the ability that ROS oxidized nonfluorescent 2,7-dichlorofluorescin diacetate (DCFH-DA) into fluorescent dichlorofluorescein (DCF). PVECs were stained with DCFH-DA (10 μmol/l, Nanjing Jiancheng Bioengineering Institute, China) at 37 °C for 30 min. Subsequently, the intracellular ROS were evaluated through fluorescence microscopy (Leica, Germany).

### Immunoelectron microscopy

The mice lung tissue was processed for electron microscope pre-embedding immunogold labeling as we previously described [19]. A Tom70 primary antibody (1:100, Sigma-Aldrich, USA) was used for immunocytochemical staining of 50-μm-thick lung tissue sections.

### Measurements of hemodynamics

Mice were lightly anesthetized with 3% isoflurane. Right ventricular systolic pressure (RVSP) was measured by right heart catheterization using Millar pressure transducer catheter. The catheter was inserted into the right jugular vein, advanced into superior vena cava, and finally into RV. After measurement of RVSP, the thorax was opened and the heart was dissected. And the weight ratio of the right ventricle (RV) divided by the sum of left ventricle (LV) and septum (S) (RV/(LV + S)) was determined as an index for RV hypertrophy. After measurement, the lung tissues were harvested for subsequent experiments.

### Morphological investigation

The right lungs were placed in neutral buffer (pH 7.4) containing 10% formalin for 72 h embedded in paraffin, sectioned into 5 μm thick sections and then subjected to hematoxylin and eosin staining. Microscopic evaluation showed structure remodeling of the pulmonary vessel. Total 80 of pulmonary vessel in approximate round shape were obtained from each group. Their external diameters from 30–60 μm were measured by an image-processing program (Image-Pro Plus, Version 5.1, Media Cybernetics, USA). The medial wall thickness, the cross-sectional area of medial wall, and the total cross-sectional vessel area were obtained. Pulmonary vascular structure remodeling was assessed by percent medial wall thickness (MT%) and percent medial wall area (MA%) two indices: MT% = 100 × (2 × medial wall thickness)/(vessel diameter), MA% = 100 × (cross sectional medial wall area)/(total cross-sectional vessel area). All the morphological analysis was conducted via a double-blind method.

### Statistical analysis

SPSS 22.0 for Windows (SPSS Inc., Chicago, IL) was used to conduct statistical analysis, and the values were expressed as means ± SD. One-way ANOVA was used for multiple group comparisons, and between-group differences were detected with post hoc Student–Newman–Keuls tests. Unpaired t test was used for two group comparisons. *P* < 0.05 indicated statistical significance.

## Results

### Identifications for the classification and purity of PVECs

**Fig. 1:**
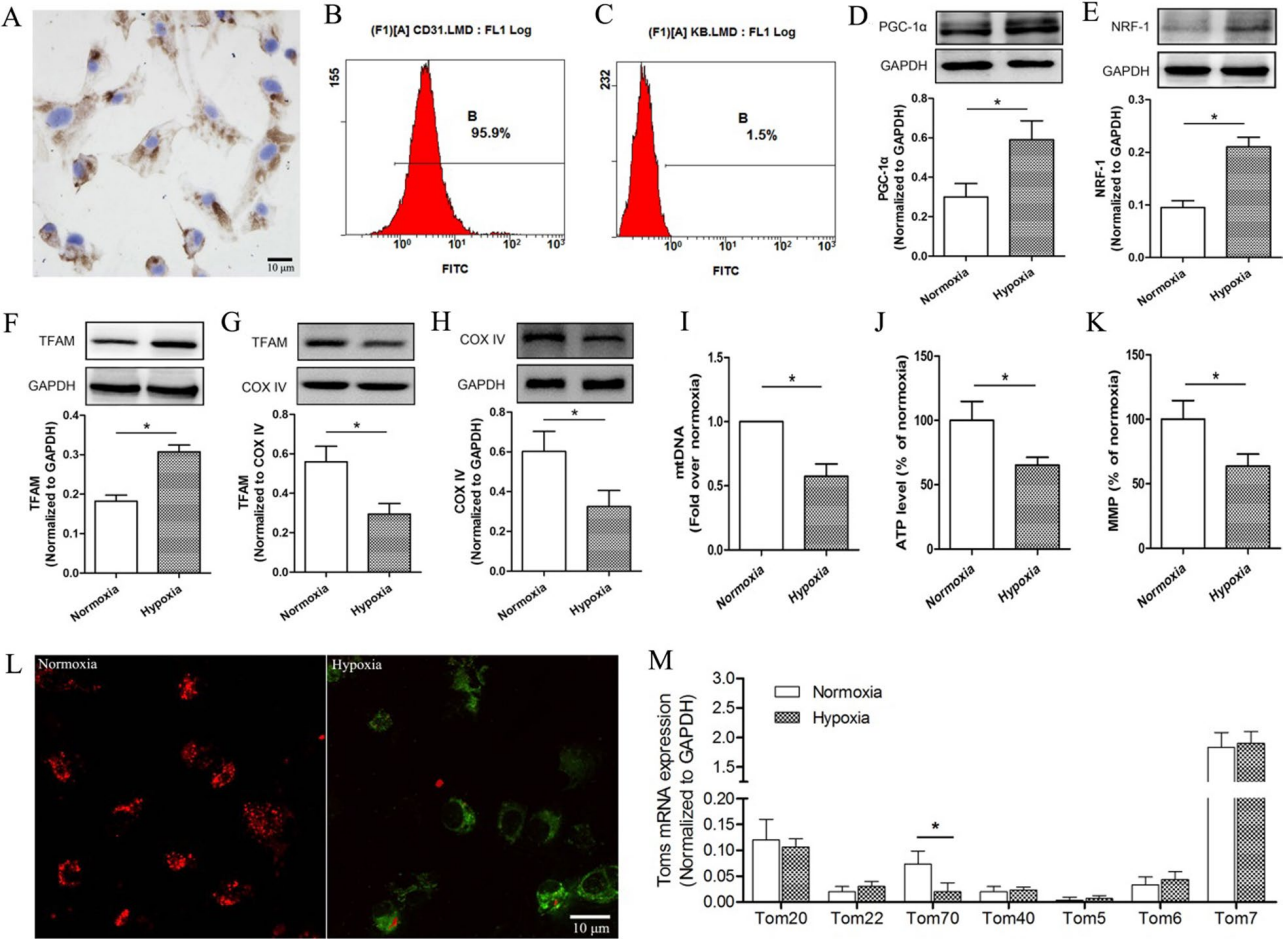
Effects of hypoxia on the level of mitochondrial biogenesis and the expressions of TOM components in PVECs at 24 h after hypoxia. To identify the classification and purity of PVECs, CD31 immunohistochemical staining (**A**) and flow cytometry (**B**) were detected. **C** Negative control for flow cytometry detection. **D**–**F** Hypoxia increased the expressions of PGC-1α, NRF-1 and TFAM. **G** Hypoxia decreased the content of TFAM in mitochondria. **H** and **I** The levels of mitochondrial complex IV (COX IV) and mitochondrial DNA (mtDNA) were reduced at 24 h after hypoxia. **J**–**L** The ATP levels and mitochondrial membrane potential (MMP) were decreased at 24 h after hypoxia. **M** The components expressions of mitochondrial outer membrane translocatase (TOM) were detected in primary cultured PVECs at 24 h after hypoxia, including Tom20, Tom22, Tom70, Tom40, Tom5, Tom6 and Tom7. *PVECs* pulmonary vascular endothelial cells, n = 4, Scale bar = 10 μm. Data are means ± SD. *P < 0.05

We detected the classification and purity of PVECs respectively by CD31 immunohistochemical staining and flow cytometry. Approximately 96% of the cells in such cultures were shown to be PVECs based on the characterizations (Fig. 1A–C).

### Effects of hypoxia on the level of mitochondrial biogenesis in PVECs

To investigate the effects of hypoxia on mitochondrial biogenesis of PVECs, we detected the expressions of mitochondrial biogenesis markers, TFAM level inside mitochondria, mitochondrial quantity and function in primary cultured PVECs at 24 h after hypoxia. Compared with the normoxia group, we found that hypoxia increased the expressions of PGC-1α, NRF-1 and TFAM (Fig. 1D–F), but decreased the content of TFAM in mitochondria (Fig. 1G). After hypoxia, the levels of mitochondrial complex IV (COX IV) and mitochondrial DNA (mtDNA) were reduced compared with the normoxia group (Fig. 1H, I), meanwhile, the mitochondrial membrane potential (MMP) and ATP levels were decreased obviously (Fig. 1J–L).

### Effects of hypoxia on the expressions of TOM components in PVECs

**Fig. 2:**
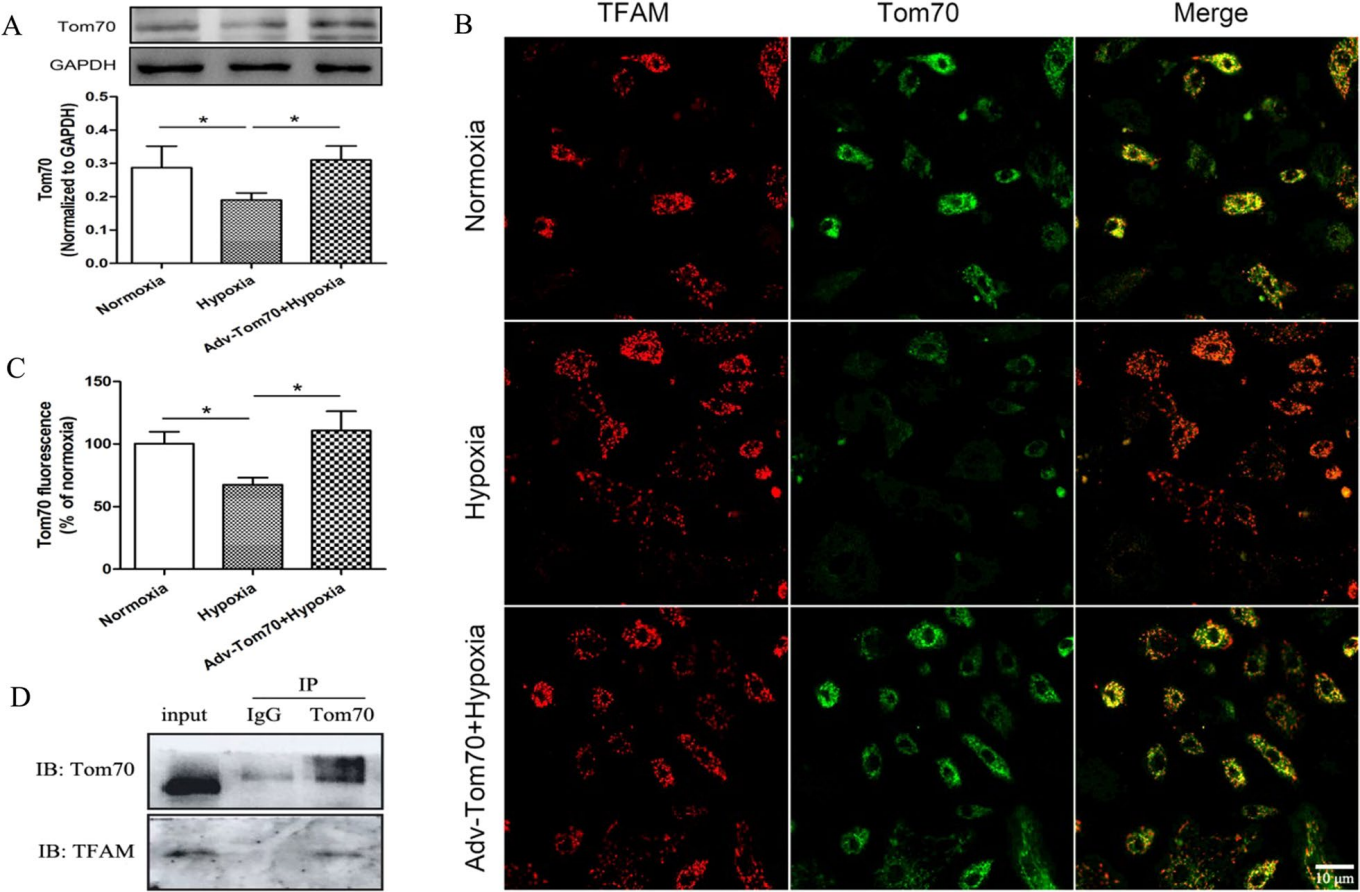
The expression of Tom70 in PVECs after hypoxia. **A** Western blot analysis was taken to assess Tom70 protein expression. **B** and **C** Immunofluorescence analysis was taken to assess Tom70 expression and co-localization between Tom70 and TFAM. **D** Co-immunoprecipitation analysis was taken to assess the interaction between Tom70 and TFAM. The expression of Tom70 was decreased at 24 h after hypoxia, and Tom70 overexpression adenovirus could increase Tom70 expression after hypoxia. *PVECs* pulmonary vascular endothelial cells, *Adv-Tom70* Tom70 overexpression adenovirus, *IP* immunoprecipitation, *IB* immunoblotting, n = 4, Scale bar = 10 μm. Data are means ± SD. *P < 0.05

At 24 h after hypoxia, the components expressions of mitochondrial outer membrane translocatase (TOM) were detected in primary cultured PVECs, including Tom20, Tom22, Tom70, Tom40, Tom5, Tom6 and Tom7. We found that only Tom70 mRNA level was decreased significantly compared with the normoxia group after hypoxia (Fig. 1M). To further observe the Tom70 expression after hypoxia, Western blotting and immunohistochemistry were used to detect the Tom70 protein expression. We found that the protein expression of Tom70 was reduced significantly compared with the normoxia group (Fig. 2A–C).

### Effects of Tom70 on the expressions of mitochondrial biogenesis markers and mitochondrial TFAM in PVECs after hypoxia

**Fig. 3:**
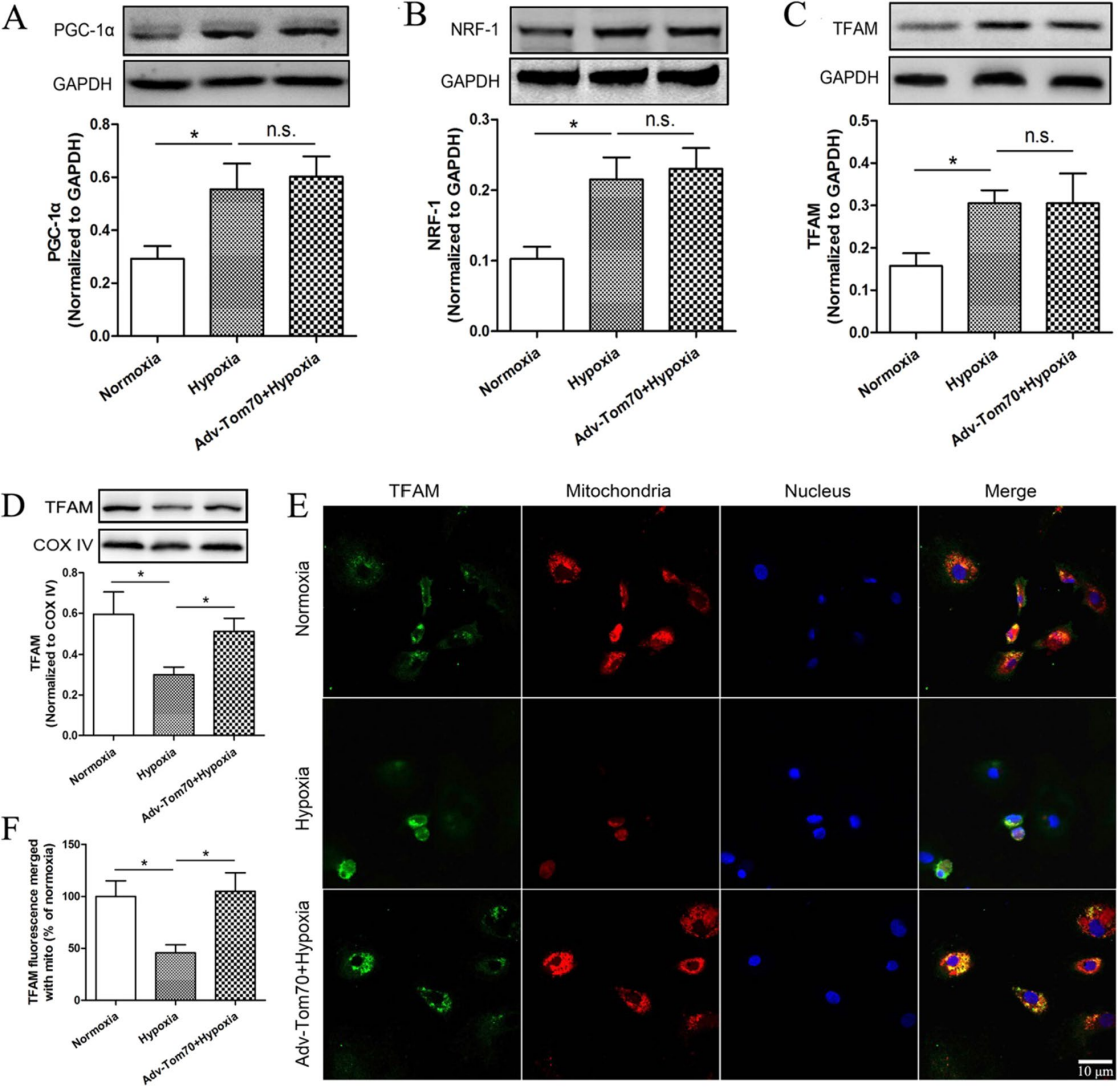
Effects of Tom70 on the expressions of mitochondrial biogenesis markers in PVECs after hypoxia. **A**–**C** Up-regulation of Tom70 had no effects on the expressions of PGC-1α, NRF-1 and TFAM. **D** Western blot analysis showed that the content of TFAM in mitochondria was reduced at 24 h after hypoxia, and up-regulation of Tom70 increased the content of TFAM in mitochondria of PVECs. **E** Immunofluorescence analysis showed the content of TFAM in mitochondria of PVECs. **F** Quantitative analysis for the TFAM fluorescence merged with mitochondria. *PVECs* pulmonary vascular endothelial cells, *mito* mitochondria, n = 4, Scale bar = 10 μm. Data are means ± SD. *P < 0.05. n.s. no significance

To explore the effects of Tom70 on mitochondrial biogenesis of PVECs after hypoxia, Tom70 overexpression adenovirus was constructed. We found that Tom70 overexpression adenovirus increased the Tom70 protein expression after hypoxia (Fig. 2A–C). Compared with the hypoxia group, up-regulation of Tom70 had no effects on the expressions of PGC-1α, NRF-1 and TFAM (Fig. 3A–C), but increased the content of TFAM in mitochondria (Fig. 3D–F). Meanwhile, there are co-localization and interaction between Tom70 and TFAM (Fig. 2B, D). These findings above indicated that Tom70 might mediate the transport of TFAM into mitochondria in primary cultured PVECs.

### Effects of Tom70 on the mitochondrial quantity, volume, and function in PVECs after hypoxia

**Fig. 4:**
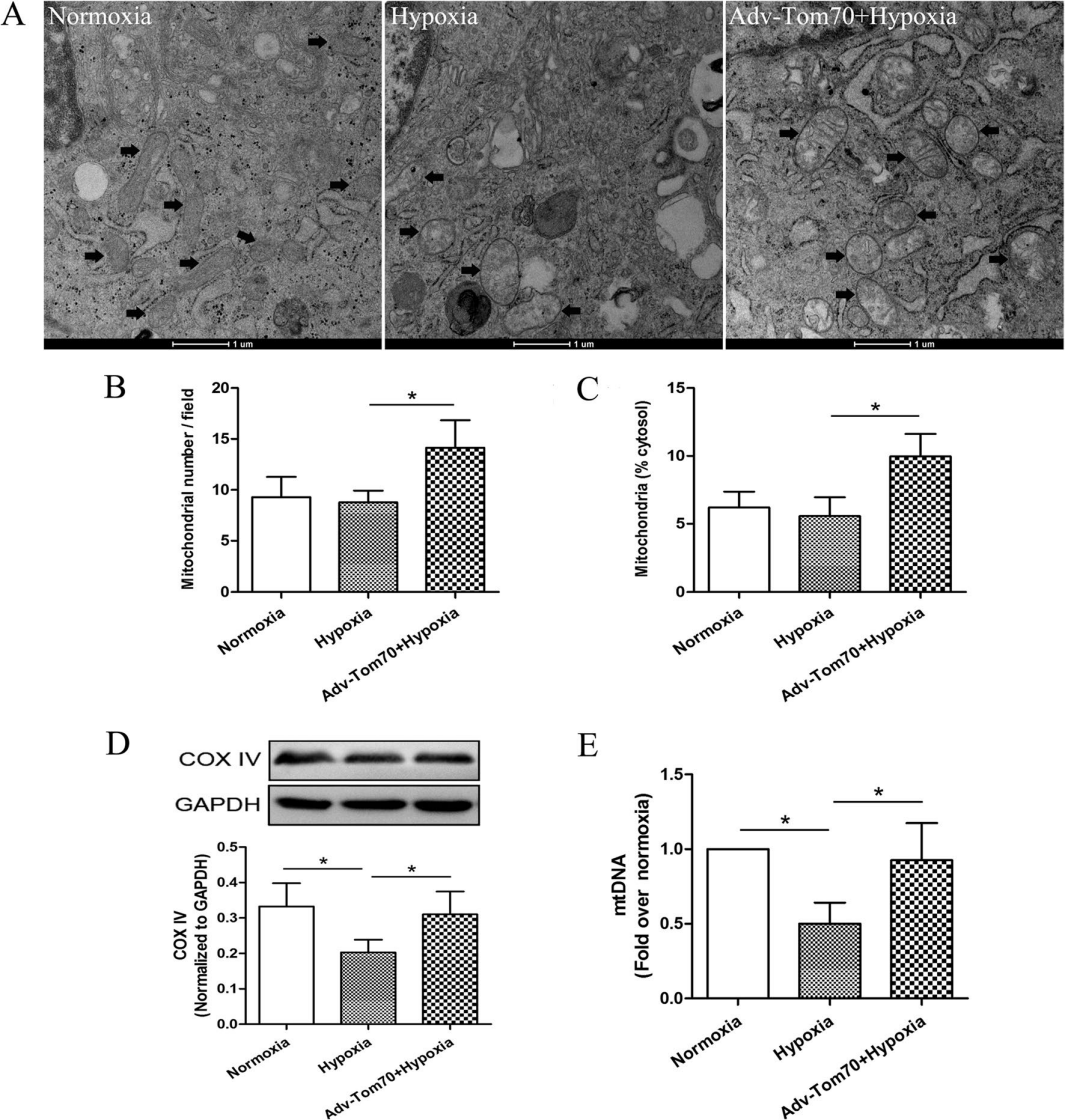
Effects of Tom70 on the mitochondrial quantity and volume in PVECs after hypoxia. **A**–**C** Up-regulation of Tom70 enhanced the mitochondrial counting (indicated by black arrows) and percentage in cytosol by electronic microscope. **D** and **E** Up-regulation of Tom70 increased the levels of mitochondrial complex IV (COX IV) and mitochondrial DNA (mtDNA) after hypoxia. *PVECs* pulmonary vascular endothelial cells, *Adv-Tom70* Tom70 overexpression adenovirus, n = 4, Scale bar = 1 μm. Data are means ± SD. *P < 0.05

**Fig. 5:**
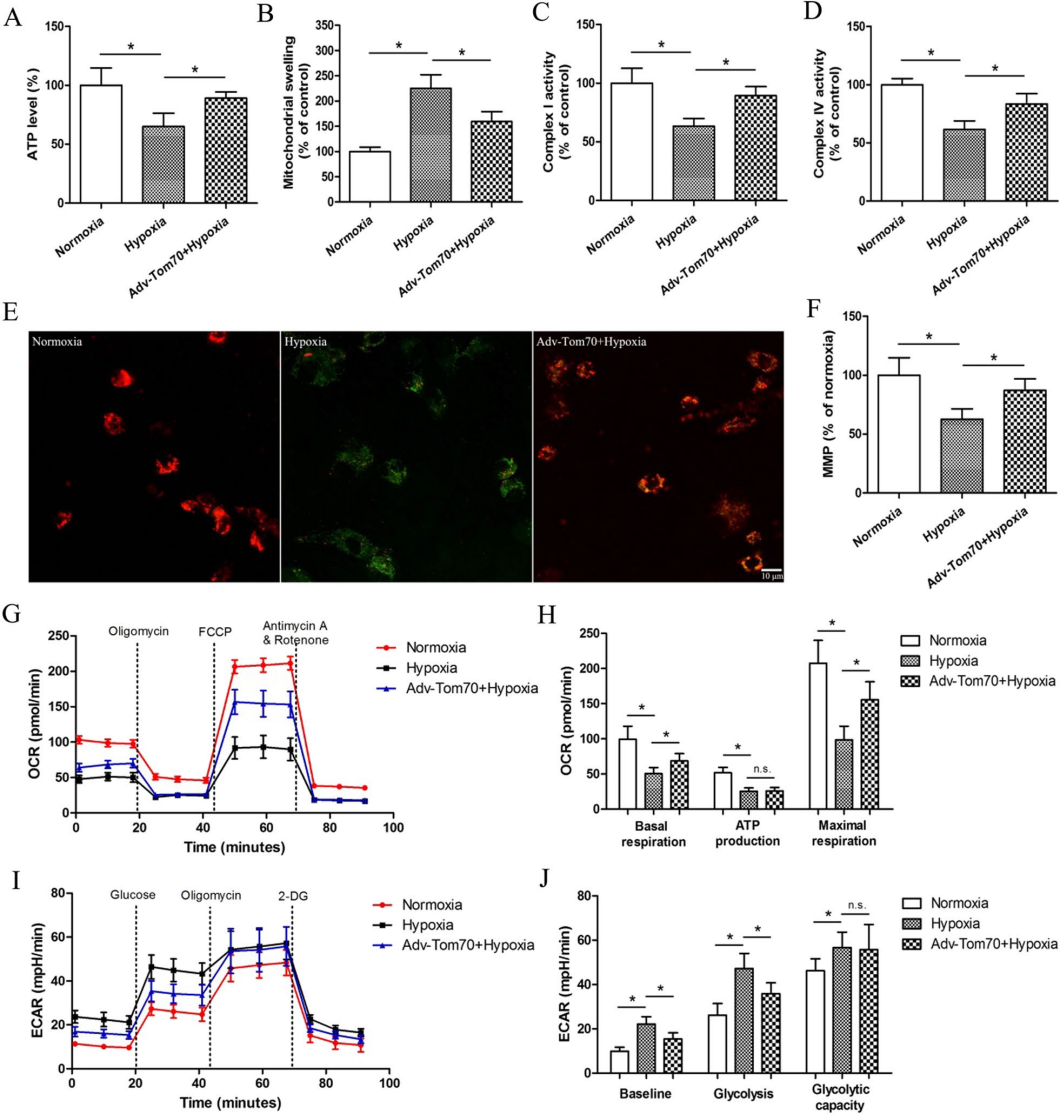
Effects of Tom70 on the mitochondrial function in PVECs after hypoxia. The ATP level (**A**), mitochondrial complex I and IV activities (**C**, **D**) and MMP level (**E**, **F**) were decreased, and the mitochondrial swelling degree was increased after hypoxia (**B**). Up-regulation of Tom70 reversed the hypoxia-induced effects on the mitochondrial functions. The baseline, ATP-production and maximal respiration rates were decreased (**G**, **H**) and the basal glycolysis, glycolysis and oligomycin-induced maximal glycolysis were increased (**I**, **J**) after hypoxia by using a Seahorse analyzer. Tom70 up-regulation partially abolished the hypoxia-induced effects on the basal and maximal respiration (**G**, **H**), and the basal glycolysis and glycolysis (**I**, **J**). *Adv-Tom70* Tom70 overexpression adenovirus, *MMP* mitochondrial membrane potential, *OCR* oxygen consumption rate, *ECAR* extracellular acidification rate, n = 4, Scale bar = 10 μm. Data are means ± SD. *P < 0.05

At 24 h after hypoxia treatment, electronic microscope, Western blotting and real-time PCR results showed that mitochondrial biomarker COX IV and mtDNA level were significantly decreased (Fig. 4D, E), and Tom70 overexpression adenovirus obviously enhanced the mitochondrial quantity, mitochondrial percentage, COX IV and mtDNA level (Fig. 4A–E). Moreover, to further explore the effects of Tom70 on mitochondrial biogenesis, we measured the mitochondrial functions of PVECs. We found that the mitochondrial swelling degree was increased dramatically after hypoxia compared with the normoxia group (Fig. 5B). Meanwhile, the ATP level (Fig. 5A), mitochondrial complex I and IV activities (Fig. 5C, D) and MMP level (Fig. 5E, F) were decreased obviously in the hypoxia group compared with the normoxia group. However, Tom70 overexpression adenovirus markedly reversed the hypoxia-induced effects on the mitochondrial functions. Seahorse analysis showed that Tom70 up-regulation increased the basal and maximal respiration rates (Fig. 5G, H), and decreased the basal glycolysis and glycolysis (Fig. 5I, J) after hypoxia. These findings indicated that up-regulation of Tom70 could enhance mitochondrial biogenesis in primary cultured PVECs.

### Effects of Tom70 on the functions of PVECs after hypoxia

**Fig. 6:**
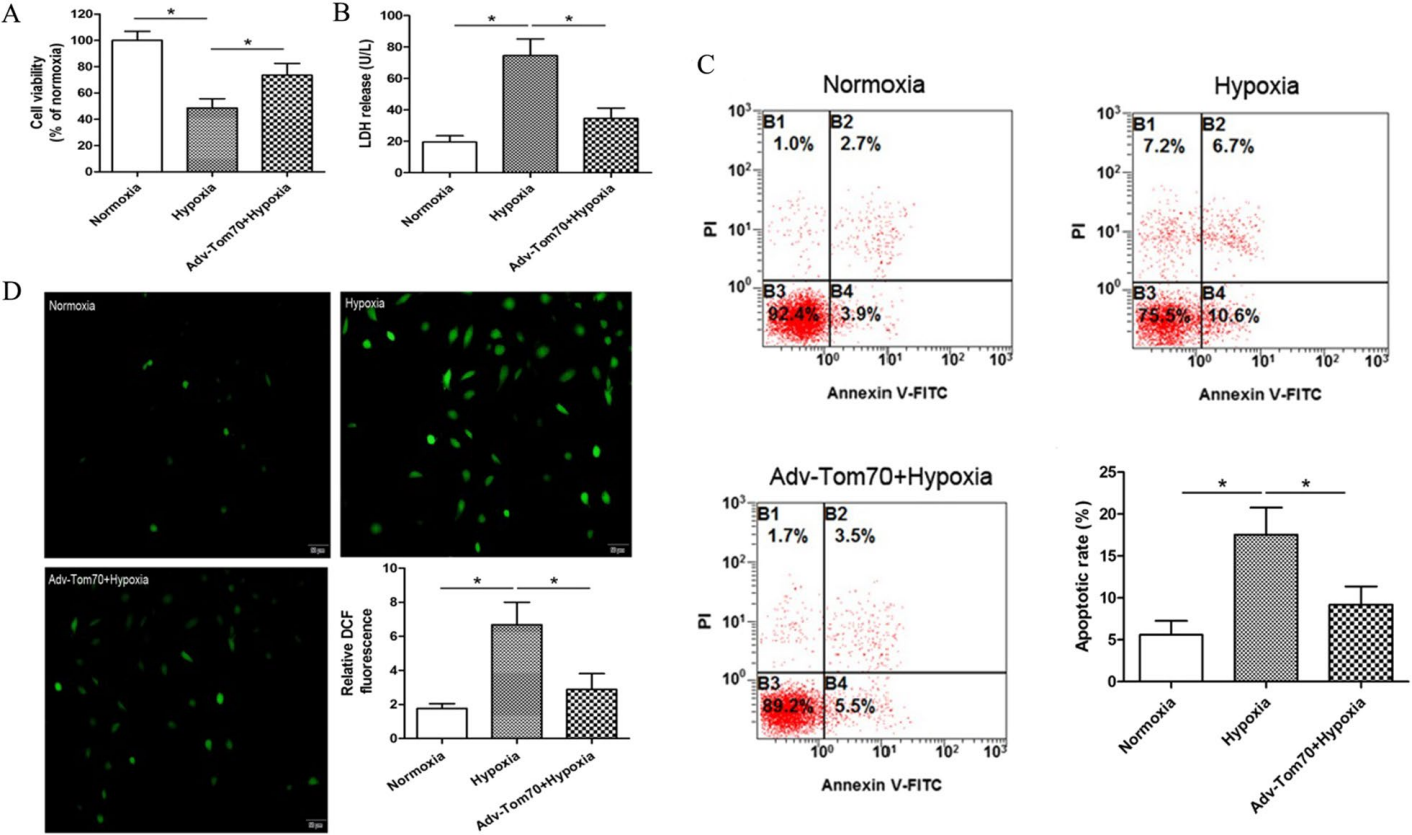
Effects of Tom70 on the functions of PVECs after hypoxia. Hypoxia reduced cell viability assessed by WST-8 assay (**A**), increased the LDH release, cell apoptosis and ROS level (**B**–**D**). Up-regulation of Tom70 partially abolished the hypoxia-induced PVECs injuries. *PVECs* pulmonary vascular endothelial cells, *Adv-Tom70* Tom70 overexpression adenovirus, *LDH* lactate dehydrogenase, n = 8, Scale bar = 50 μm. Data are means ± SD. *P < 0.05

To further observe the effects of Tom70 on the functions of PVECs after hypoxia, we took primary cultured PVECs hypoxia model. Compared with the normoxia group, we found that hypoxia reduced cell viability assessed by WST-8 assay (Fig. 6A), increased the LDH release, cell apoptosis and ROS level (Fig. 6B–D). And up-regulation of Tom70 partially abolished the hypoxia-induced PVECs injury above.

### Beneficial effects of PVECs Tom70 up-regulation on HPH in vivo

**Fig. 7:**
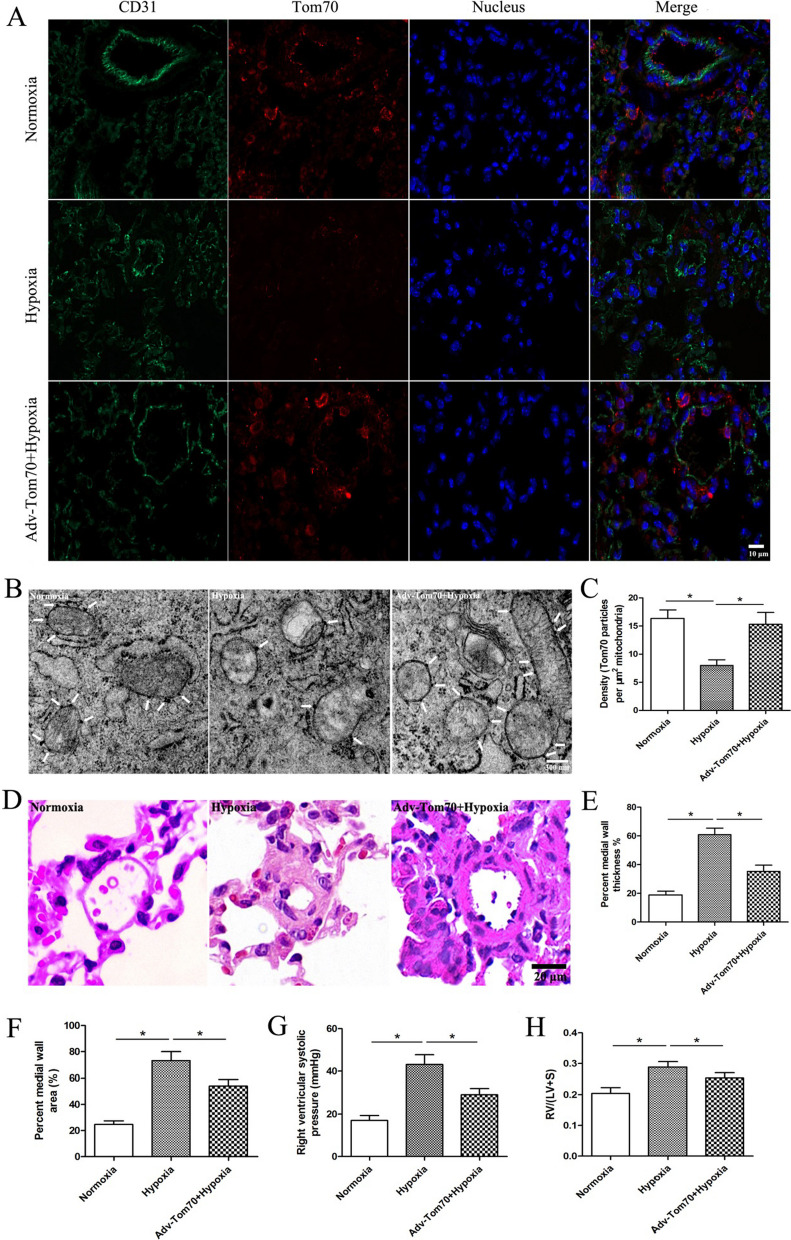
Beneficial effects of PVECs Tom70 up-regulation on HPH in vivo. **A** Immunofluorescence showed the Tom70 expression in PVECs, n = 3, Scale bar = 10 μm. **B** Immunoelectron microscopy showed the Tom70 expression in PVECs (indicated by white arrows), Scale bar = 300 nm. **C** Density of Tom70 immunoparticles in PVECs were calculated per area of mitochondria, n = 3. **D** Hematoxylin and eosin staining of pulmonary vessels, Scale bar = 20 μm. **E**–**H** Hypoxia increased MT%, MA%, RVSP and RV/(LV + S), and up-regulation of Tom70 in PVECs partially reversed the hypoxia-induced injurious effects, n = 8. *PVECs* pulmonary vascular endothelial cells, *Adv-Tom70* Tom70 overexpression adenovirus, *MT%* percent medial wall thickness, *MA%* percent medial wall area, *RVSP* right ventricular systolic pressure, *RV* right ventricle; *LV* left ventricle; *S* septum. Data are means ± SD. *P < 0.05

To further verify the effects of Tom70 on HPH, we used cre-dependent overexpression adenovirus of Tom70 in the Cdh5-CreERT2 mouse model of HPH to selectively upregulating the expression of Tom70 in PVECs. Immunofluorescence and immunoelectron microscopy were used to detect the Tom70 expression. We found that hypoxia reduced the expression of Tom70 in PVECs compared with the normoxia group, while Tom70 overexpression adenovirus increased the Tom70 expression in PVECs after hypoxia (Fig. 7A–C). The hematoxylin and eosin staining of lung tissue and the summarized data of MT% and MA% of pulmonary artery are shown in Fig. 7D–F, respectively, which showed that hypoxia significantly increased MT% and MA% compared with the normoxia group. Meanwhile, hypoxia markedly increased the RVSP (Fig. 7G) and RV/(LV + S) (Fig. 7H). However, Tom70 up-regulation in PVECs partially reversed the hypoxia-induced injurious effects above.

## Discussion

**Fig. 8:**
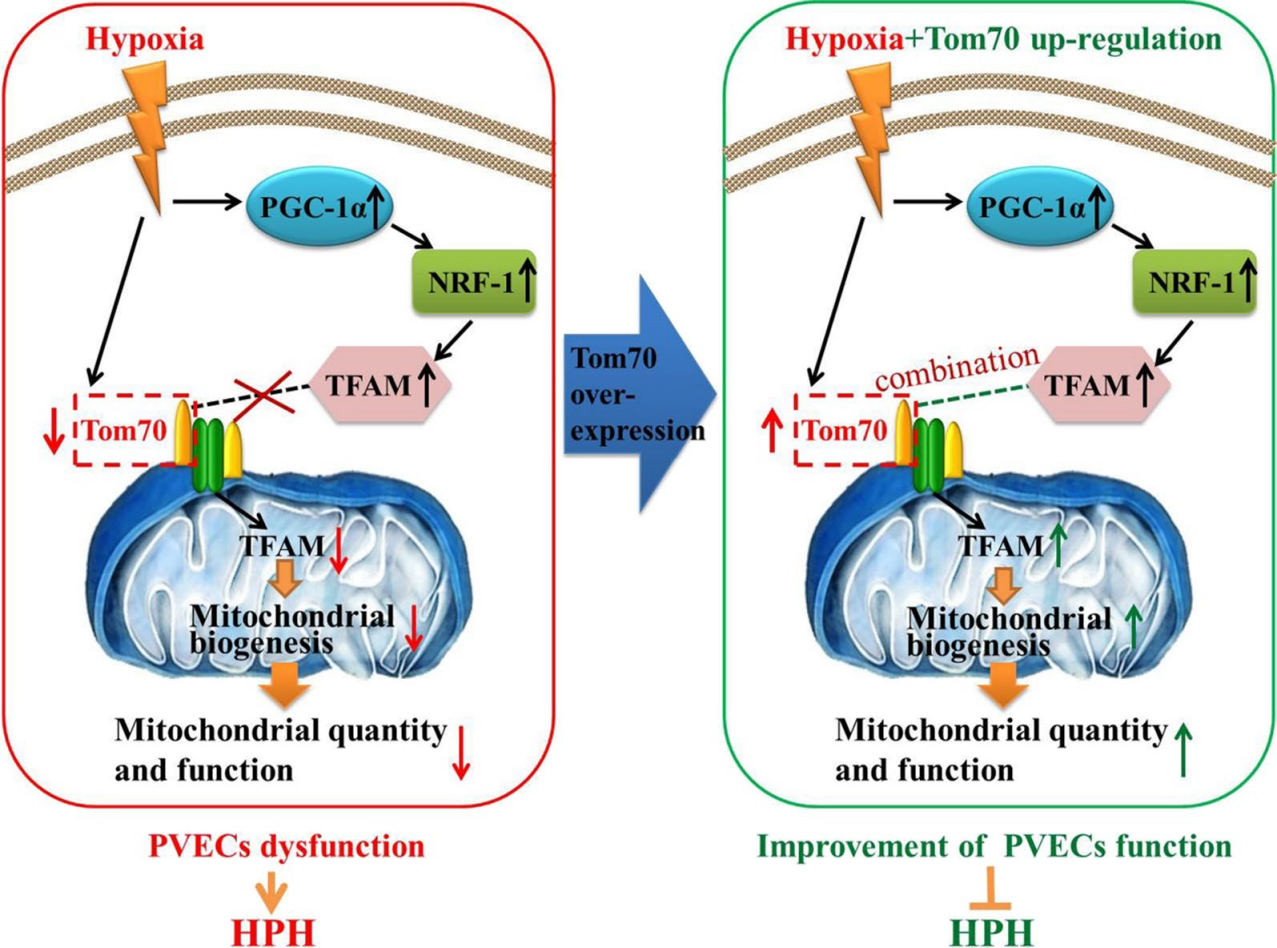
Working model illustrates that hypoxia induced the decreased expression of Tom70 in PVECs, reduced the mitochondrial biogenesis-associated TFAM protein transporting into mitochondria, inhibited mitochondrial biogenesis, caused PVECs dysfunction, and prompted the formation of HPH. However, up-regulation of Tom70 abolished the hypoxia-induced injurious effects in PVECs and alleviated HPH. *PVECs* pulmonary vascular endothelial cells, *HPH* hypoxic pulmonary hypertension

In this investigation, we found that hypoxia obviously increased the expressions of PGC-1α, NRF-1 and TFAM, but decreased the content of TFAM in mitochondria and the quantity and functions of mitochondria. In addition, only Tom70 expression among the TOM components was significantly decreased after hypoxia, and up-regulation of Tom70 significantly increased the content of TFAM in mitochondria of PVECs by transporting TFAM into mitochondria after hypoxia, enhanced the quantity and functions of mitochondria, improved the functions of PVECs, and ultimately alleviated HPH. These results indicated that Tom70-regulated mitochondrial biogenesis via TFAM might play an important role in improving mitochondrial functions of PVECs, reducing PVECs injury and alleviating HPH after hypoxia (Fig. 8).

Numerous studies have shown that pulmonary endothelial injury is the primary factor causing hypoxic pulmonary vasoconstriction and pulmonary vascular structural remodeling, and playing an important role in the pathophysiological processes of HPH [5, 7]. Nitric oxide (NO), the endothelium-derived relaxing factor, is an important vasodilator produced by endothelial cells, which can inhibit the contraction of vascular smooth muscle and the proliferation of smooth muscle cells [13]. While hypoxia can also cause contraction of vascular smooth muscle by reducing the production and bioavailability of NO [20]. Reactive oxygen species (ROS) are mainly produced by mitochondria, and serve as a signaling molecule to involve in cell proliferation, differentiation and apoptosis [21]. Under pathological conditions such as hypoxia, the mitochondrial function of endothelial cells is impaired, leading to the production of a large amount of ROS. The accumulation of ROS within cells can induce lipid peroxidation and the opening of mitochondrial permeability transition pores, eventually causing endothelial cells damage [22, 23]. Some studies showed that during the occurrence of pulmonary arterial hypertension, the quantity and function of mitochondria and mtDNA in PVECs decreased, and a large amount of ROS is produced, leading to PVECs injury [24, 25], which are consistent with our findings.

Some studies have indicated that mitochondria are constantly undergoing dynamic changes, including mitochondrial biogenesis, autophagy, fusion and fission [26, 27]. Mitochondrial biogenesis, an endogenous cytoprotective response, refers to the process by which cells increase the quantity and function of mitochondria, representing an attempt by cells to increase or maintain their previous functional state when faced with a decrease in mitochondrial quantity or function [28]. In this study, we found that the expressions of PGC-1α, NRF-1 and TFAM, the key transcription factors for mitochondrial biogenesis, were significantly increased after hypoxia, indicating that hypoxia induced the activation of mitochondrial biogenesis pathway in PVECs. The results are consistent with previous research findings [29]. However, the functional TFAM within mitochondria for mitochondrial biogenesis were reduced, resulting in a decrease in the quantity and function of mitochondria. Therefore, we speculated that there must be some mechanism that blocks the improvement of mitochondrial quantity and function after the activation of mitochondrial biogenesis pathway.

In the process of mitochondrial biogenesis, the synthesized precursor proteins need to be transported into mitochondria through the translocase of outer mitochondrial membrane (TOM), and then perform biological functions [30, 31]. The TOM complex consists of a central channel composed of Tom40, Tom5, Tom6 and Tom7, and three receptor proteins including Tom70, Tom20 and Tom22. Receptor proteins are responsible for recognizing mitochondrial localization signals and transporting these precursor proteins to the central channel of mitochondria [32–34]. The TOM, also known as the mitochondrial gate, plays a crucial role in mitochondrial biogenesis [35]. Previous studies have found that abnormalities in the main components of TOM proteins are involved in the occurrence and development of various diseases [36, 37]. However, how to change in the TOM complex of PVECs after hypoxia is still not clear. In this study, we detected the components expressions of TOM complex in PVECs after hypoxia treatment and found that only Tom70 level were significantly reduced compared with normoxia. While up-regulating Tom70 expression increased the TFAM content in mitochondria of PVECs after hypoxia, enhanced mitochondrial quantity and functions, and ultimately improved the functions of PVECs. Moreover, other studies have found that the mitochondrial damage caused by abnormal transport of PINK1 into mitochondria mediated by Tom70 is a key factor in the occurrence and development of Parkinson's disease [36]. In the study of cold-stress, it was also found that mitochondrial function is impaired due to abnormal protein transport mediated by Tom70 into mitochondria [37]. In addition, the interaction between Tom70 and TFAM in cultured retinal endothelial cells in vitro was confirmed by co-immunoprecipitation [38], which was also demonstrated in this study by the methods of fluorescence co-localization and co-immunoprecipitation. These results showed that Tom70 might promote mitochondrial biogenesis of PVECs after hypoxia by transporting TFAM into mitochondria, thereby improving PVECs function.

From the perspective of mitochondrial material transport, our findings revealed that the decreased expression of Tom70 after hypoxia leads to abnormal transport of the key factor TFAM into mitochondria, which is the crucial mechanism for the obstruction of mitochondrial biogenesis pathway and PVECs injury after hypoxia. Our findings also offered a novel therapeutic target for the treatment of HPH. However, there are still some limitations in our investigation. At present, we can only increase the expression of Tom70 through overexpression, which is difficult to be applied in clinical studies. A recent study showed that melatonin could improve mitochondrial function by increasing Tom70 expression [39]. Therefore, we will explore whether melatonin can improve PVECs function after hypoxia and alleviate HPH by up-regulating Tom70, providing theoretical basis for the clinical application of melatonin in the treatment of HPH.

## Conclusion

Our study determined that hypoxia induced the decreased expression of Tom70 in PVECs, reduced the mitochondrial biogenesis-associated TFAM protein transporting into mitochondria, inhibited mitochondrial biogenesis, caused PVECs injury, and prompted the formation of HPH. However, up-regulation of Tom70 abolished the hypoxia-induced injurious effects on PVECs and alleviated HPH.

### Supplementary Information

**Additional file 1. **Original gel and blot images.

## Data Availability

The datasets used and/or analyzed during the current study are available from the corresponding author on reasonable request.

## Abbreviations

HPH: Hypoxic pulmonary hypertension
PVECs: Pulmonary vascular endothelial cells
TOM: Translocase of outer mitochondrial membrane
TFAM: Mitochondrial transcription factor A
NRF-1: Nuclear respiratory factor 1
PGC-1α: Peroxisome proliferator-activated receptor (PPAR)-γ coactivator-1α
CMS: Chronic mountain sickness
COPD: Chronic obstructive pulmonary disease
ROS: Reactive oxygen species
NO: Nitric oxide
mtDNA: Mitochondrial DNA
COX: Mitochondrial complex
MMP: Mitochondrial membrane potential
LDH: Lactate dehydrogenase
OCR: Oxygen consumption rate
ECAR: Extracellular acidification rate
MT%: Percent medial wall thickness
MA%: Percent medial wall area
RVSP: Right ventricular systolic pressure
RV: Right ventricle
LV: Left ventricle
S: Septum
