# Supplementary figures and images for: Tom70-regulated mitochondrial biogenesis via TFAM improves hypoxia-induced dysfunction of pulmonary vascular endothelial cells and alleviates hypoxic pulmonary hypertension
Source: Respir Res. 2023 Dec 13;24:310. doi: 10.1186/s12931-023-02631-y (PMC10717060; doi:10.1186/s12931-023-02631-y)

**Fig. 1D**

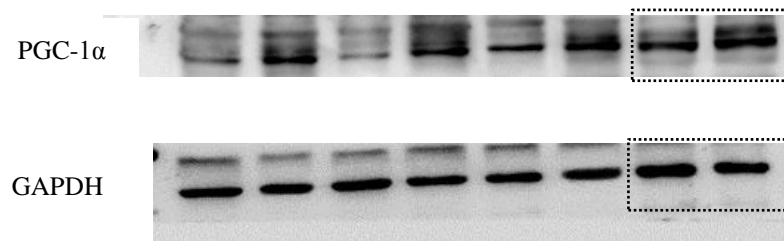

**Fig. 1E**

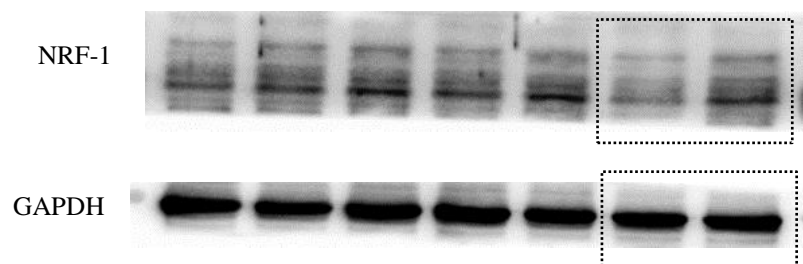

**Fig. 1F**

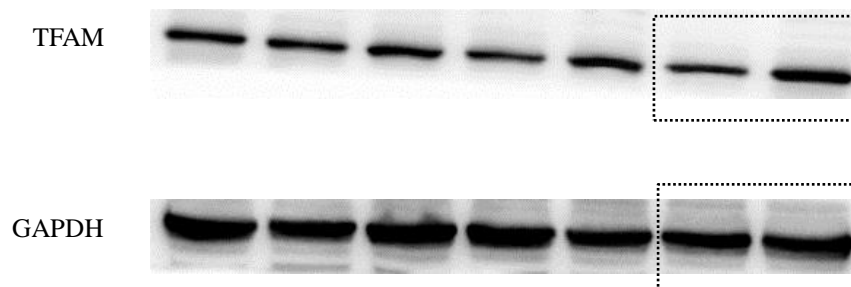

**Fig. 1G**

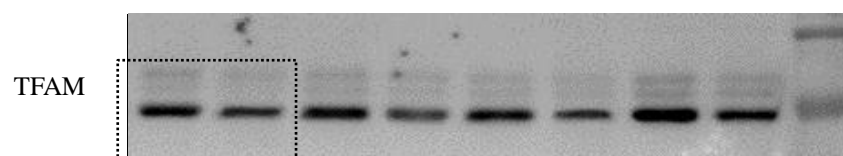

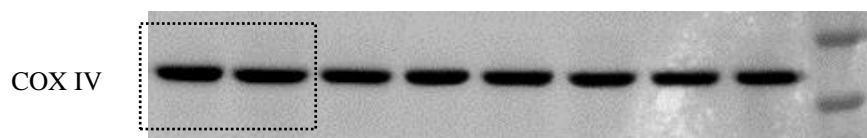

**Fig. 1H**

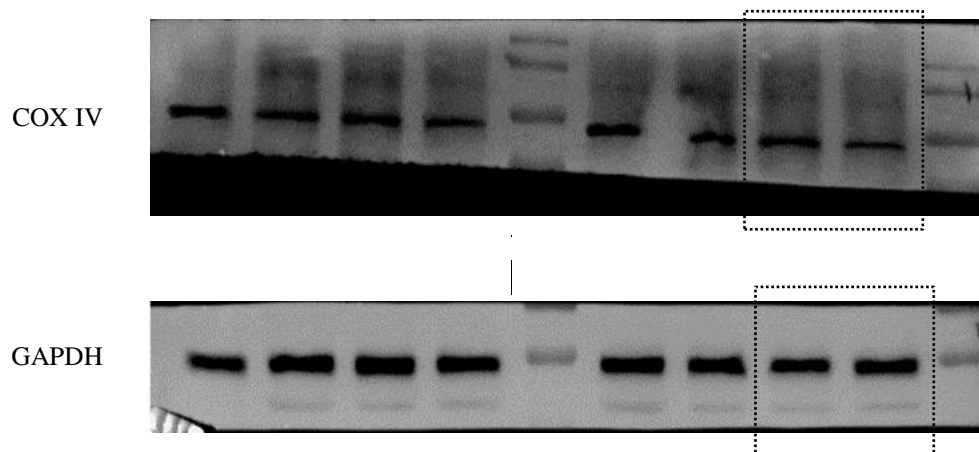

**Fig. 2A**

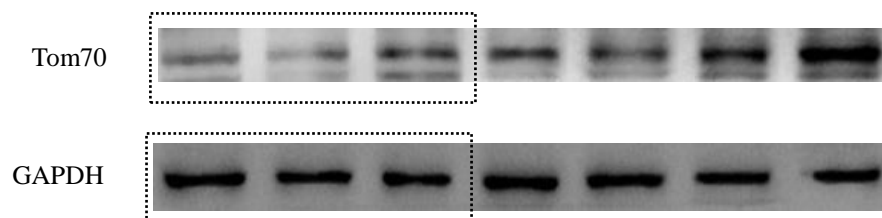

**Fig. 2D**

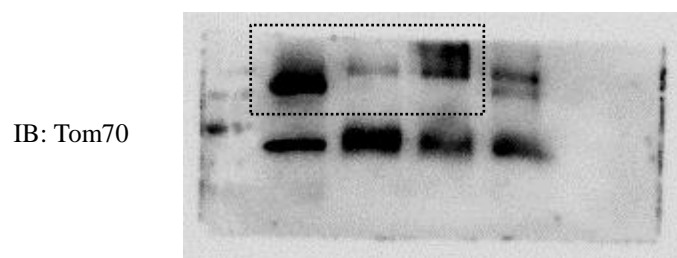

IB: TFAM

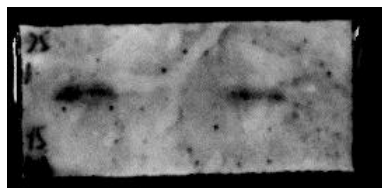

**Fig. 3A**

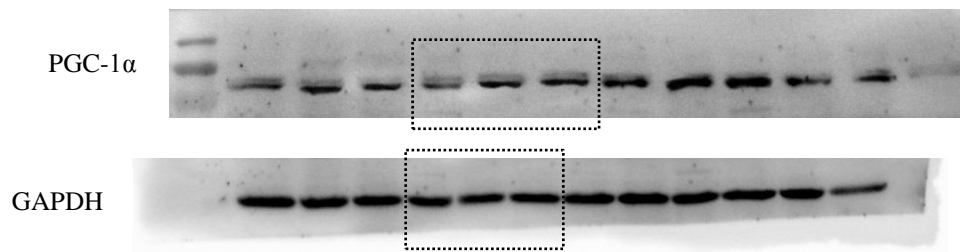

**Fig. 3B**

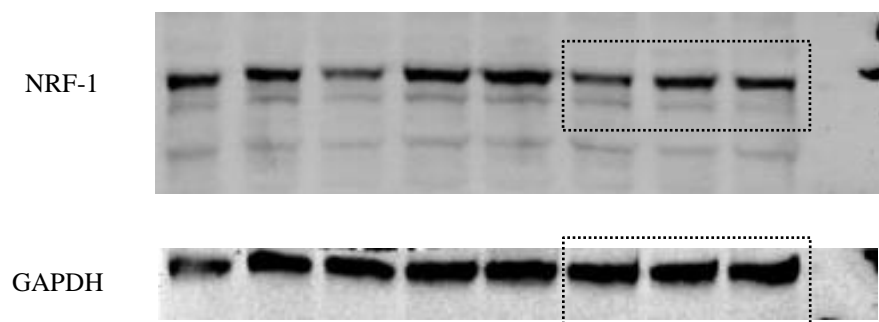

**Fig. 3C**

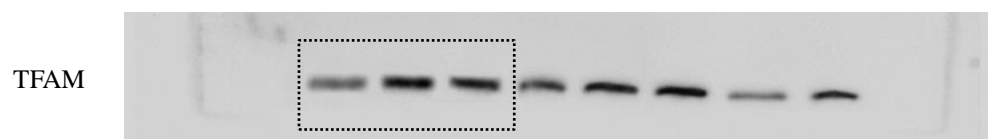

GAPDH

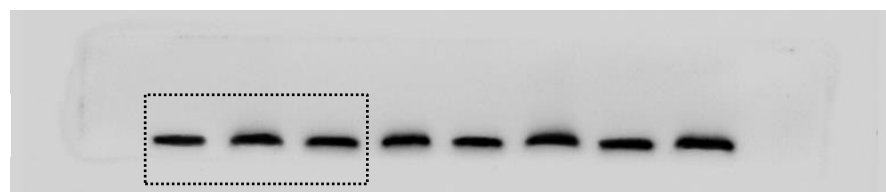

**Fig. 3D**

TFAM

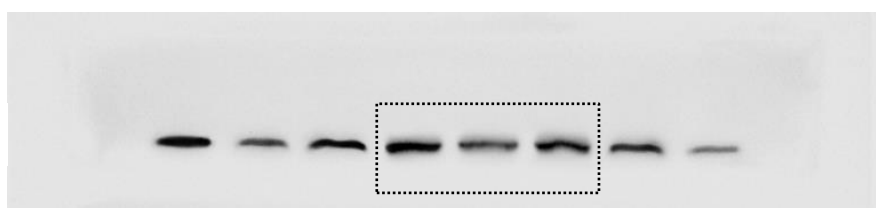

COX IV

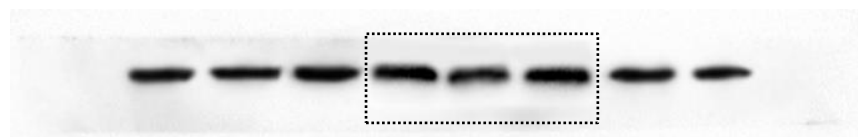

**Fig. 4D**

COX IV

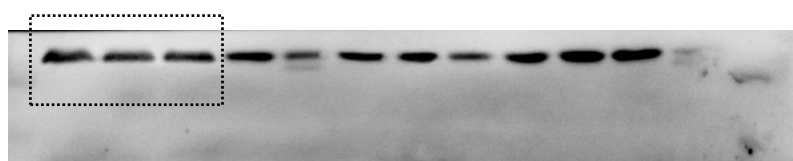

GAPDH

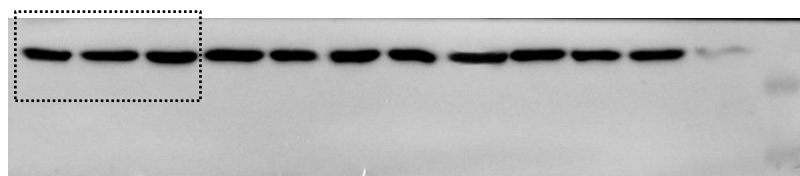

Supplement: Supplementary file 1 — Additional file 1. Original gel and blot images. [file 12931_2023_2631_MOESM1_ESM.pdf]
